# The RNA-binding protein IMP2 drives a stromal-Th17 cell circuit in autoimmune neuroinflammation

**DOI:** 10.1172/jci.insight.152766

**Published:** 2022-02-08

**Authors:** Rami Bechara, Nilesh Amatya, Saikat Majumder, Chunsheng Zhou, Yang Li, Qixing Liu, Mandy J. McGeachy, Sarah L. Gaffen

**Affiliations:** 1 Université Paris-Saclay, CEA, INSERM UMR 1184, Center for Research in Immunology of Viral, Autoimmune, Hematological and Bacterial Diseases (IMVA-HB), Le Kremlin Bicêtre, France.; 2 Division of Rheumatology and Clinical Immunology, Department of Medicine, University of Pittsburgh, Pittsburgh, Pennsylvania, USA.; 3 Tsinghua University, Beijing, China.

**Keywords:** Autoimmunity, Immunology, Chemokines, Cytokines, Signal transduction

## Abstract

Stromal cells are emerging as key drivers of autoimmunity, partially because they produce inflammatory chemokines that orchestrate inflammation. Chemokine expression is regulated transcriptionally but also through posttranscriptional mechanisms, the specific drivers of which are still incompletely defined. CCL2 (MCP1) is a multifunctional chemokine that drives myeloid cell recruitment. During experimental autoimmune encephalomyelitis (EAE), an IL-17–driven model of multiple sclerosis, CCL2 produced by lymph node (LN) stromal cells was essential for immunopathology. Here, we showed that *Ccl2* mRNA upregulation in human stromal fibroblasts in response to IL-17 required the RNA-binding protein IGF-2 mRNA-binding protein 2 (IGF2BP2, IMP2), which is expressed almost exclusively in nonhematopoietic cells. IMP2 binds directly to *CCL2* mRNA, markedly extending its transcript half-life, and is thus required for efficient CCL2 secretion. Consistent with this, *Imp2*^−/−^ mice showed reduced CCL2 production in LNs during EAE, causing impairments in monocyte recruitment and Th17 cell polarization. *Imp2*^–/–^ mice were fully protected from CNS inflammation. Moreover, deletion of IMP2 after EAE onset was sufficient to mitigate disease severity. These data showed that posttranscriptional control of *Ccl2* in stromal cells by IMP2 was required to permit IL-17–driven progression of EAE pathogenesis.

## Graphical abstract

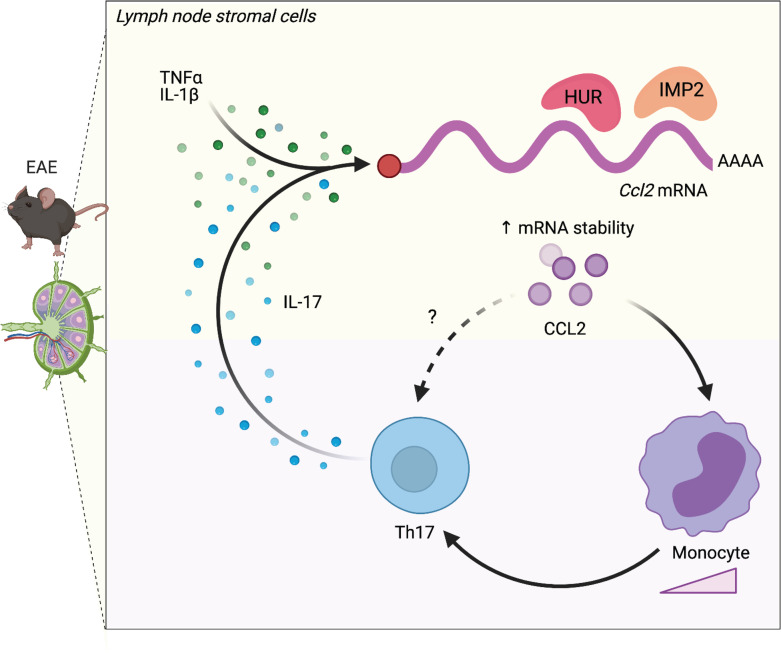

## Introduction

Autoimmune diseases encompass a spectrum of disorders characterized by aberrant immune response to self-antigens, usually of unknown etiology. Despite substantial advances in anticytokine biologic drugs, there is a major unmet need for additional effective treatments for autoimmune conditions. Achieving this goal will require a better understanding of the molecular pathways and mechanisms that promote autoimmunity.

Multiple sclerosis is a T cell–mediated demyelinating condition of the CNS. Experimental autoimmune encephalomyelitis (EAE) is a widely used mouse model that has shaped our understanding of the pathogenesis of multiple sclerosis and many other autoimmune conditions. This model has been the impetus for effective therapeutic approaches for multiple sclerosis, particularly the discovery of the Th17 pathway and inhibitors thereof (1). During EAE, the generation of encephalitogenic Th17 cells is dependent on myelin antigen processing and presentation.

CCL2 (MCP1) is a key player in driving EAE pathogenesis (2, 3). Deletion of *Ccl2* or its receptor CCR2 led to reduced CNS inflammation in EAE (4–7). In addition to governing leukocyte movement, CCL2 has been suggested to directly regulate T cell priming and polarization in draining lymph nodes (LNs) (2, 8). Indeed, crosstalk between stromal cells and lymphocytes is becoming increasingly appreciated, and in EAE, LN stromal cells express high levels of CCL2 and actively mediate immune responses (9). Hence, CCL2 acts to trigger chemotaxis and may also sustain T cell differentiation and effector functions.

At homeostasis, the *Ccl2* gene is transcribed at low tonic levels but is rapidly induced after exposure to inflammatory stimuli, notably by the encephalitogenic cytokine IL-17A (here termed IL-17) (10–12). IL-17 family cytokines comprise a structurally and biochemically distinct class of factors with distinct signaling mechanisms compared with other inflammatory stimuli. Consequently, IL-17–driven events are still poorly defined. In recent years, it has become increasingly evident that IL-17 upregulates downstream target genes in large part through posttranscriptional control of mRNA, which is orchestrated by a highly complex but still poorly understood network of RNA-binding proteins (RBPs) (13). Although transcriptional induction of *Ccl2* at the level of its promoter is well defined (14), far less is known about posttranscriptional regulation mechanisms, which are essential to determine overall mRNA levels (15).

The RBP IMP2 (also known as IGF-2 mRNA-binding protein 2, IGF2BP2) has been implicated in control of cellular metabolism and tumor progression. However, IMP2 is not detectably highly expressed in most hematopoietic cells, and accordingly, until recently it has been overlooked in the context of the immune system (16, 17). We recently discovered a key role for IMP2 in driving a mouse model of autoantibody-induced glomerulonephritis (AGN). In AGN, IMP2 promotes expression of CCAAT/enhancer binding protein (C/EBP) transcription factors in the IL-17 and TNF signaling pathways and drives downstream expression of renal-damaging effector genes (18). The role of IMP2 in other autoimmune settings remains unknown.

Here, we showed that IMP2 is essential for expression of CCL2 in human stromal fibroblasts, which we demonstrated in human LN stromal cells and in mouse embryonic fibroblasts (MEFs). IMP2 functions by binding directly to *Ccl2* mRNA, prolonging *Ccl2* transcript half-life and enhancing CCL2 secretion from stromal cells. During EAE, IMP2 is essential for inducible expression of CCL2 within the LNs. Consequently, loss of IMP2 leads to impaired monocyte accumulation in LNs and concomitantly impaired Th17 cell differentiation during EAE. Thus, *Imp2*^–/–^ mice are fully resistant to EAE, indicating that control of CCL2 expression at a posttranscriptional level is required for autoimmune disease progression. Moreover, inducible loss of IMP2 after initiation of EAE also lessens disease. Together, these data indicate that IMP2 is at the nexus of a stromal cell/T cell autoimmune circuit that drives neuroinflammation through a CCL2/Th17 cell axis.

## Results

### Imp2-deficient mice are resistant to EAE.

**Figure 1:**
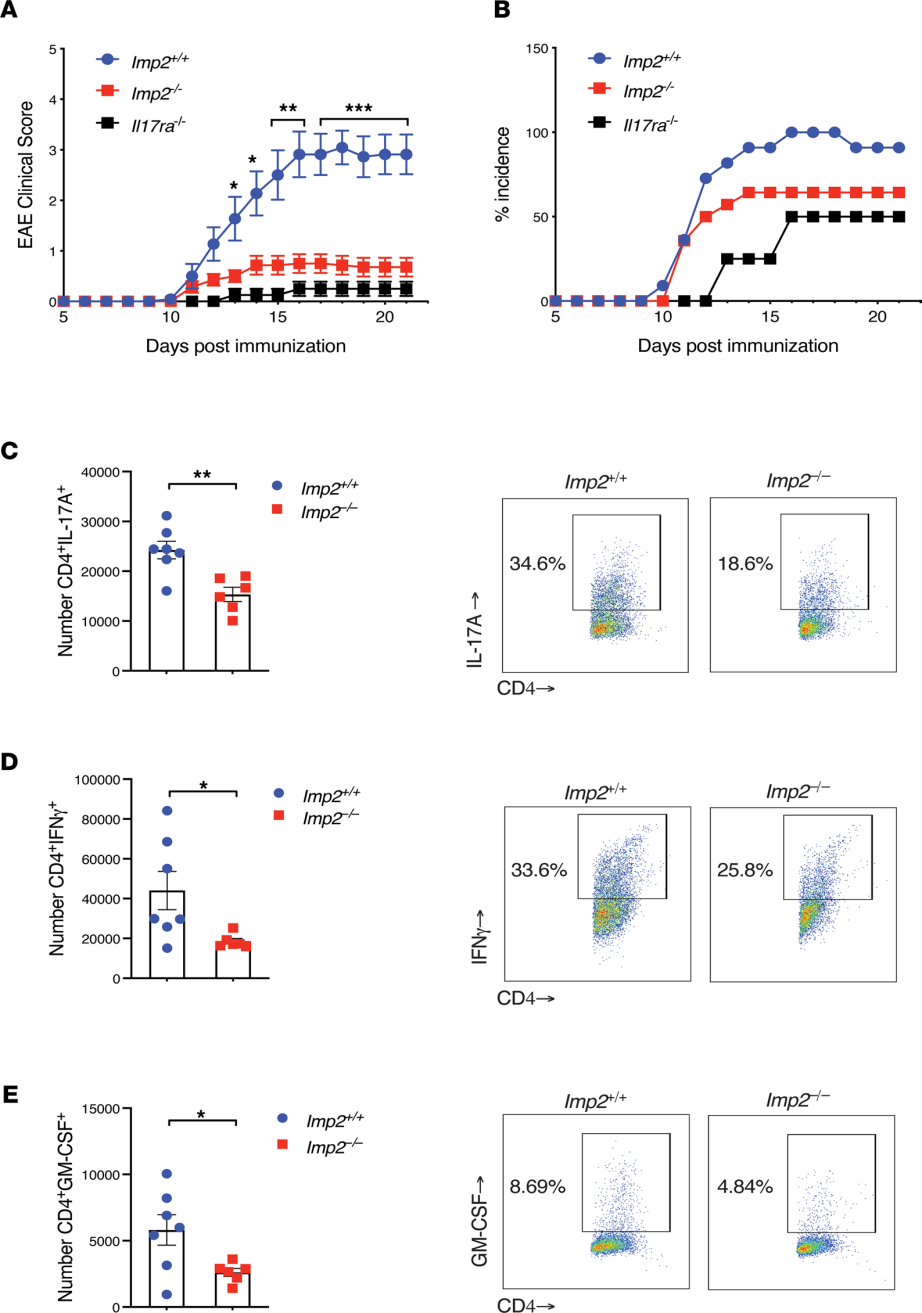
IMP2^–/–^ mice are refractory to IL-17–driven neuroinflammation. (**A**) The indicated mice (*Imp2^+/+^* *n* = 11; *Imp2*^–/–^ *n* = 14; and *Il17ra^–/–^* *n* = 4) were subjected to EAE, and clinical scores were assessed daily. Mean clinical scores pooled from 3 independent experiments are shown. (**B**) Percentage of mice exhibiting EAE symptoms (incidence). (**C**–**E**) The indicated mice were subjected to EAE (*Imp2^+/+^* *n* = 7; *Imp2*^–/–^ *n* = 6). CNS cells harvested on day 16 were treated with PMA/ionomycin for 4 hours. Cells were stained for CD4, IL-17A, GM-CSF, and IFN-γ and quantified by flow cytometry. *Right*: representative FACS plots. Data were pooled from 2 independent experiments. Throughout, each symbol represents 1 mouse. *P < 0.05, **P < 0.01, ***P < 0.001 by Mann-Whitney test (**A**) or unpaired Student’s *t* test (**C**–**E**).

To interrogate a role for IMP2 in EAE, we induced disease in mice by immunization with myelin oligodendrocyte glycoprotein peptide (MOG 35-55) in CFA. Mice were scored daily for signs of ascending paralysis. *Imp2*^+/+^ control mice developed a typical onset and clinical course of EAE, peaking at 14–16 days after immunization (Figure 1A). As previously shown, mice lacking the IL-17 receptor (*Il17ra^–/–^*) were fully resistant to signs of disease (19). Strikingly, *Imp2*^–/–^ mice showed resistance to EAE that was similar to *Il17ra^–/–^* mice (Figure 1A). Concomitant with reduced disease scores, there was marked reduction in incidence of EAE in *Imp2*^–/–^ mice (Figure 1B). Thus, IMP2 is essential for autoimmune inflammation in EAE.

Disease in EAE is mediated by the accumulation of pathogenic Th17 cells in the CNS. To determine whether IMP2 affects Th17 cell infiltration during EAE, mice were immunized with MOG, and CNS tissues harvested at day 16 were stimulated with PMA/ionomycin and stained for T cell markers and intracellular cytokines. Consistent with the observed decrease in EAE severity, *Imp2*^–/–^ mice presented decreased numbers of IL-17–, IFN-γ–, and GM-CSF–producing CD4^+^ T cells (Figure 1, C–E, and Supplemental Figure 1; supplemental material available online with this article; https://doi.org/10.1172/jci.insight.152766DS1). Together, these results indicate that IMP2 is required for pathogenic Th17 cell infiltration in the CNS and thus for EAE pathogenesis.

### Imp2-deficient mice show impaired Th17 cell generation in LNs.

**Figure 2:**
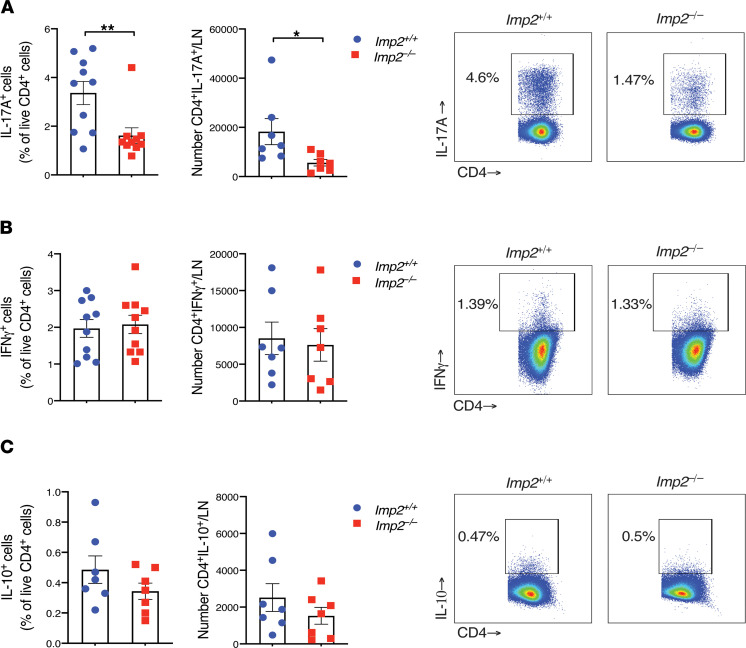
IMP2 promotes Th17 cell generation. (**A**–**C**) *Imp2*^+/+^ (*n* = 7–10) and *Imp2*^–/–^ (*n* = 7–10) mice were subjected to EAE. Inguinal lymph node cells harvested on day 10 were treated with PMA/ionomycin for 4 hours. Cells were stained for CD4, IL-17A, IL-10, and IFN-γ and quantified by flow cytometry. Data were pooled from 2–3 independent experiments. *Right*: representative FACS plots. Throughout, each symbol represents 1 mouse. *P < 0.05, **P < 0.01 by unpaired Student’s *t* test.

To understand the cause of decreased encephalitogenic Th17 cell frequencies in the setting of IMP2 deficiency, we assessed T cell differentiation in *Imp2*^–/–^ mice. Mice were immunized with MOG and draining (inguinal) LNs were harvested at day 10, stimulated with PMA/ionomycin, and stained for T cell markers and intracellular cytokines (Supplemental Figure 2). Both the percentages and total numbers of IL-17–producing T cells were reduced in *Imp2*^−/−^ mice compared with *Imp2*^+/+^ littermates (Figure 2A). In contrast, IMP2 did not affect IFN-γ or IL-10 production (Figure 2, B and C) or LN infiltration of total CD4^+^ cells (Supplemental Figure 2A). There were no baseline differences in expression of T cell cytokines in naive mice of both genotypes (Supplemental Figure 3).

### IMP2 drives Ccl2 expression and monocyte recruitment in LN.

**Figure 3:**
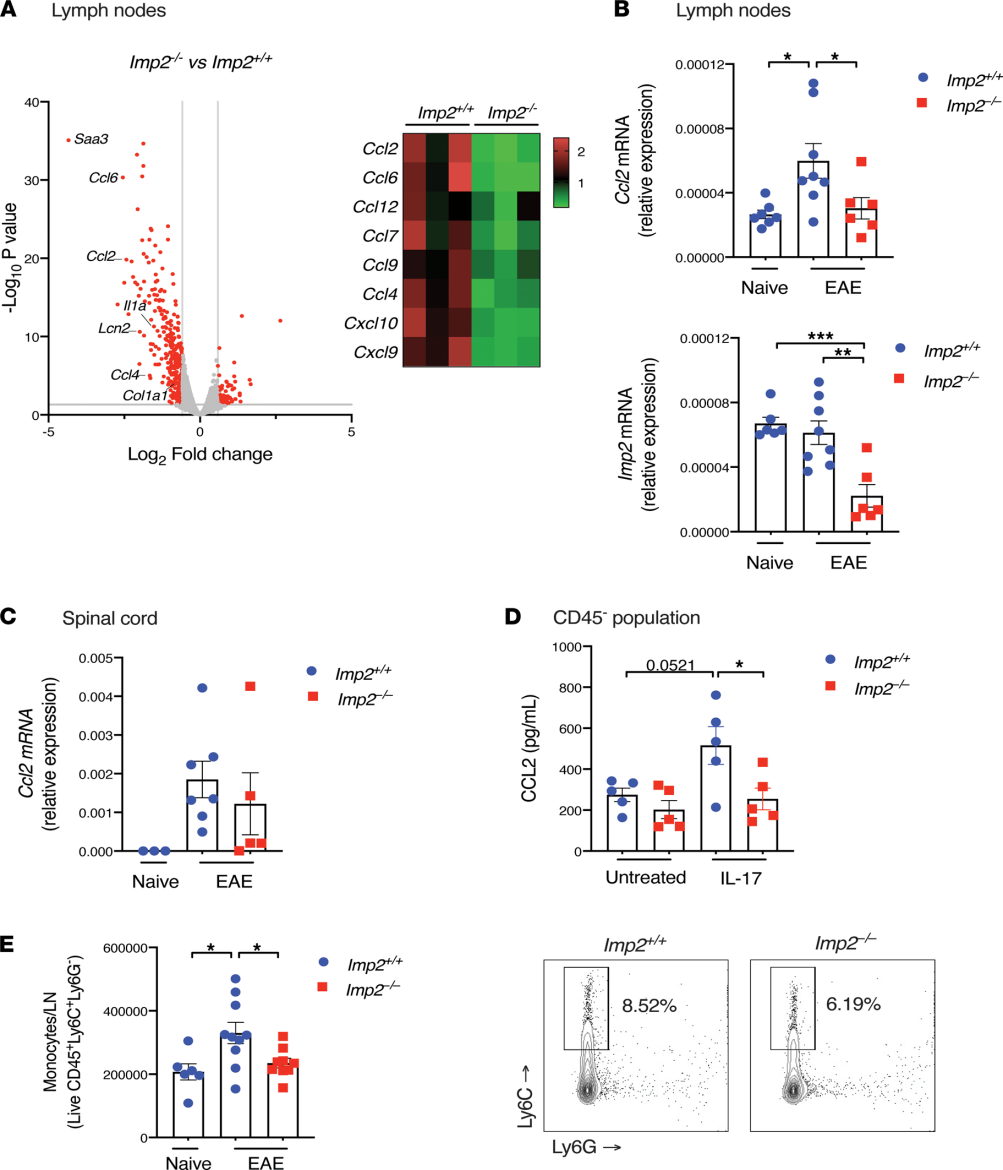
IMP2 promotes Ccl2 expression and monocyte recruitment in lymph nodes. (**A** and **B**) *Imp2*^+/+^ and *Imp2*^–/–^ mice were subjected to EAE. Inguinal LN homogenates were prepared on day 7. (**A**) RNA-Seq (*Imp2^+/+^* *n* = 3 and *Imp2*^–/–^ *n* = 3) was performed on the Illumina platform. Volcano plots showing the genes that were significantly changed (*P* < 0.05, fold change of >1.5 or <−1.5 and reads per kilobase million in *Imp2*^+/+^ > 1). Selected transcripts that are known to be IL-17 regulated are annotated. *Right*: heatmap of chemokines that are differentially regulated based on IPA. (**B**) *Imp2^+/+^* (*n* = 8) and *Imp2*^–/–^ (*n* = 6) mice were subjected to EAE. Indicated mRNAs were assessed by qPCR in inguinal LNs at day 7. Data show relative expression ± SEM from 2 independent experiments. (**C**) The indicated mice (*Imp2^+/+^* *n* = 7; *Imp2*^–/–^ *n* = 5) were subjected to EAE, and RNA from spinal cord isolated on day 15 was subjected to qPCR normalized to *Gapdh*. Data show relative expression ± SEM from 2 independent experiments. (**D**) CD45^–^ cells from *Imp2^+/+^* (*n* = 5) and *Imp2*^–/–^ (*n* = 5) mice were treated with IL-17 for 24 hours and conditioned media assessed by ELISA. Data show mean pg/mL ± SEM from 2 independent experiments. (**E**) *Imp2^+/+^* (*n* = 10) and *Imp2*^–/–^ (*n* = 9) mice were subjected to EAE. Inguinal LN homogenates were prepared on day 7. Live monocytes were determined by Ly6C and Ly6G staining, gated on the live CD45^+^ population. *Left:* numbers of live CD45^+^ Ly6C^+^Ly6G^–^ cells, pooled from 3 independent experiments. *Right*: representative FACS plots. Throughout, each symbol represents 1 mouse. *P < 0.05, **P < 0.01, ***P < 0.001, by ANOVA with post hoc Tukey’s test (**C**–**E**).

IL-17 triggers potent downstream signaling activities on LN stromal cells (20, 21). To delineate the impact of IMP2 on LNs during EAE, *Imp2^+/+^* and *Imp2*^–/–^ LNs were subjected to RNA-Seq at day 7 after EAE. A total of 383 genes were differentially regulated, and Ingenuity Pathway Analysis (IPA; QIAGEN) identified multiple differentially enriched pathways. Notably, hypercytokinemia and hyperchemokinemia were among the most altered pathways in *Imp2*^–/–^ mice (Figure 3A). Prominent among differentially expressed genes was CCL2, which is known to promote EAE by enhancing monocyte recruitment. CCL2 has also been described to promote Th17 cell polarization directly (4–8). *Imp2*^–/–^ mice showed significantly decreased expression in *Ccl2* mRNA in LNs on day 7 after EAE (Figure 3B). Although *Ccl2* was upregulated in spinal cords of mice with EAE, its expression was not statistically reduced in *Imp2*^–/–^ mice (Figure 3C). The major CCL2-expressing cells in LNs are in the nonhematopoietic (CD45^–^) stromal compartment (9), cells that are known to be responsive to IL-17 (20). To determine whether IMP2 in LN stromal cells is required to upregulate *Ccl2*, CD45^–^ populations isolated from LNs of *Imp2*^+/+^ and *Imp2*^–/–^ naive mice were treated with IL-17 for 24 hours, and CCL2 in supernatants was assessed by ELISA. CCL2 levels were elevated after IL-17 stimulation in the CD45^–^ fraction in *Imp2^+/+^* cells but were impaired in *Imp2*^–/–^ cells (Figure 3D). CCL2 was not detectable in the CD45^+^ fraction (data not shown). Together, these data suggest a stromal intrinsic role of IMP2 in driving CCL2 expression in response to IL-17.

A major function of CCL2 is to recruit monocytes to sites of inflammation. Indeed, IMP2 was required for monocyte accumulation in LNs during EAE, with *Imp2^−/−^* mice showing reduced accumulation of CD45^+^Ly6C^+^Ly6G^–^ monocytes compared with *Imp2^+/+^* controls (Figure 3E). In contrast to monocytes, *Imp2^+/+^* and *Imp2*^–/–^ mice showed similar infiltration of other myeloid cell populations during EAE, demonstrating an especially key activity of monocyte-recruiting chemoattractants such as CCL2 (Supplemental Figure 4).

**Figure 4:**
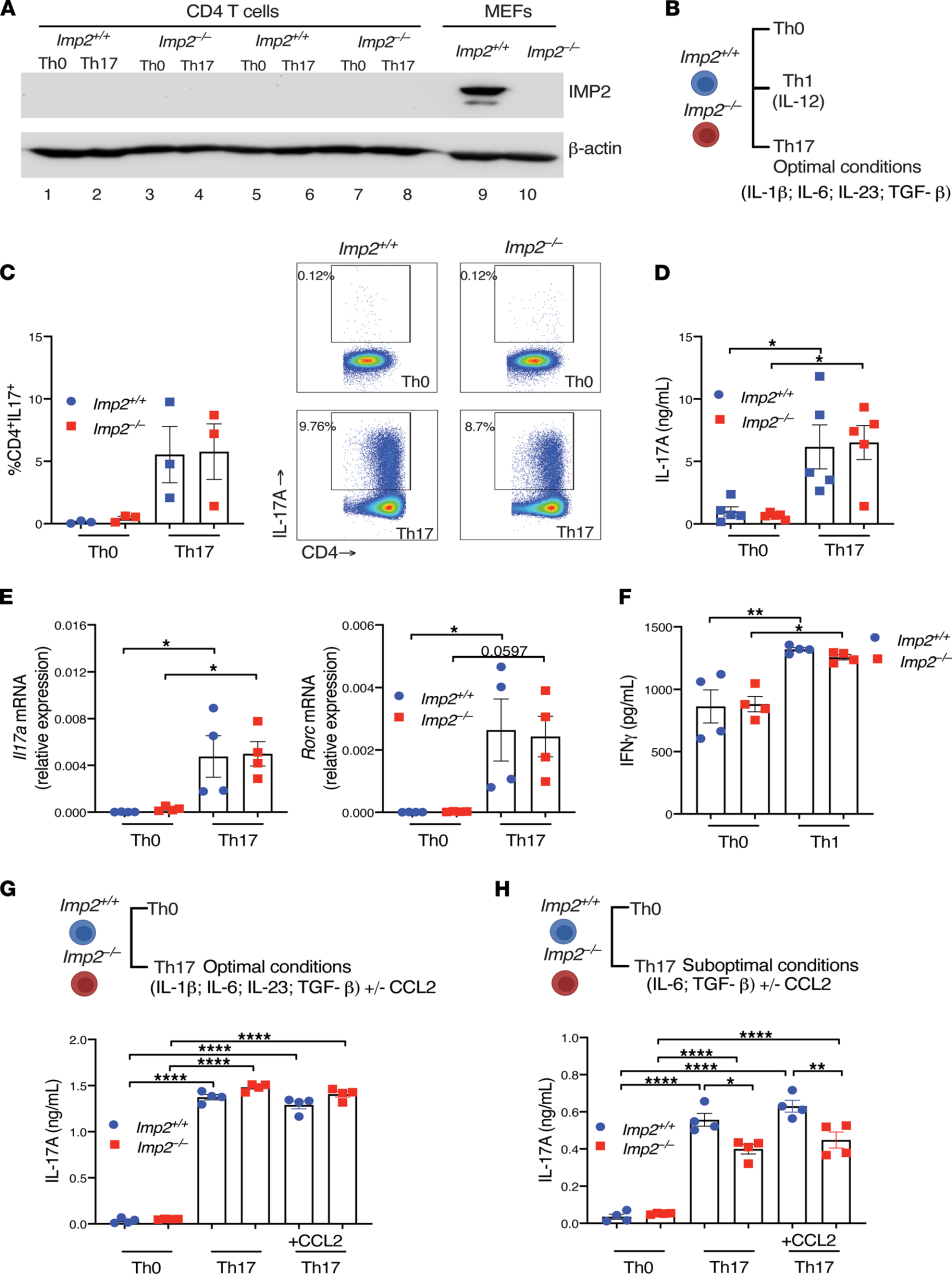
Th1- and Th17-extrinsic activities of IMP2. (**A**) IMP2 expression by Western blot on whole cell lysates from CD4^+^ T cells cultured in Th0 conditions (lanes 1, 3, 5, 7) and Th17 conditions (IL-6, IL-23, TGF-β, and IL-1β) (lanes 2, 4, 6, 8). Control lysates from *Imp2^+/+^* and *Imp2*^–/–^ MEFs (lanes 9 and 10). (**B**) Diagram of in vitro CD4^+^ T cell polarization conditions. (**C**–**E**) CD4^+^ T cells were isolated from spleen and stimulated for 3 days with plate-bound anti-CD3 and anti-CD28 under Th0 or optimal Th17 conditions (IL-6, IL-23, TGF-β, and IL-1β) for 3 days. (**C**) Cells were stimulated with PMA/ionomycin for 4 hours, stained for CD4 and IL-17, and quantified by flow cytometry. *Right*: representative FACS plots from 3 independent experiments. (**D**) ELISA was performed on supernatants collected after 3 days of treatment with anti-CD3 and anti-CD28. Data were pooled from 2 independent experiments. (**E**) qPCR of the indicated mRNAs in T cells after 3 days of anti-CD3 and anti-CD28. Data show fold change mean ± SEM from 2 independent experiments. (**F**) CD4^+^ T cells were isolated from spleen and stimulated for 3 days with plate-bound anti-CD3 and anti-CD28 under Th0 or Th1 conditions (IL-12). ELISA was performed on supernatants collected after 3 days of anti-CD3 plus anti-CD28. (**G** and **H**) CD4^+^ T cells were isolated from spleen and stimulated for 3 days with plate-bound anti-CD3 and anti-CD28 under Th0 or optimal Th17 conditions (**G**) (IL-6, IL-23, TGF-β, and IL-1β) or suboptimal Th17 conditions (**H**) (IL-6 and TGF-β) ± CCL2 for 3 days. ELISA was performed on supernatants collected after 3 days of anti-CD3 plus anti-CD28. Each symbol represents 1 mouse. *P < 0.05, **P < 0.01, ****P < 0.0001 by ANOVA with post hoc Tukey’s test.

According to the ImmGen database (https://www.immgen.org/), IMP2 is expressed at extremely low levels in most immune cells. Indeed, IMP2 was not detectable in CD4^+^ T cells either in Th0 or Th17 conditions (Figure 4A). To determine whether there is a T cell–intrinsic role for IMP2 in Th17 or Th1 differentiation, naive CD4^+^ T cells from *Imp2^+/+^* and *Imp2*^–/–^ mice were stimulated for 3 days with plate-bound anti-CD3 and anti-CD28 under Th0 or standard Th17 conditions (IL-6, TGF-β, IL-1β, and IL-23) or Th1 conditions (IL-12) (Figure 4B). As shown, *Imp2* deficiency did not affect the percentages of in vitro–generated CD4^+^IL-17^+^ Th17 cells, IL-17 production, or *Il17a* and *Rorc* expression (Figure 4, C–E). Similarly, IMP2 did not affect Th1 differentiation (Figure 4F). Thus, T cells from *Imp2*^–/–^ mice are not inherently defective in the ability to differentiate to the Th17 or Th1 lineage, at least when using optimal polarizing cytokine conditions (Figure 4B).

In addition to governing leukocyte movement, CCL2 is reported to promote development of polarized Th17 responses, and CCR2 deficiency in T cells causes decreased Th17 cell frequencies in vivo (8). However, we found that adding CCL2 to either optimal or suboptimal Th17 polarizing conditions did not affect IL-17 production (Figure 4, G and H). Interestingly, in suboptimal conditions, there was a modest but significant decrease in IL-17 production in *Imp2*^–/–^ CD4^+^ T cells, suggesting there may be subtle T cell–intrinsic capabilities of this RBP, which will need further investigation (Figure 4H).

### IMP2 mediates posttranscriptional regulation of Ccl2 mRNA.

**Figure 5:**
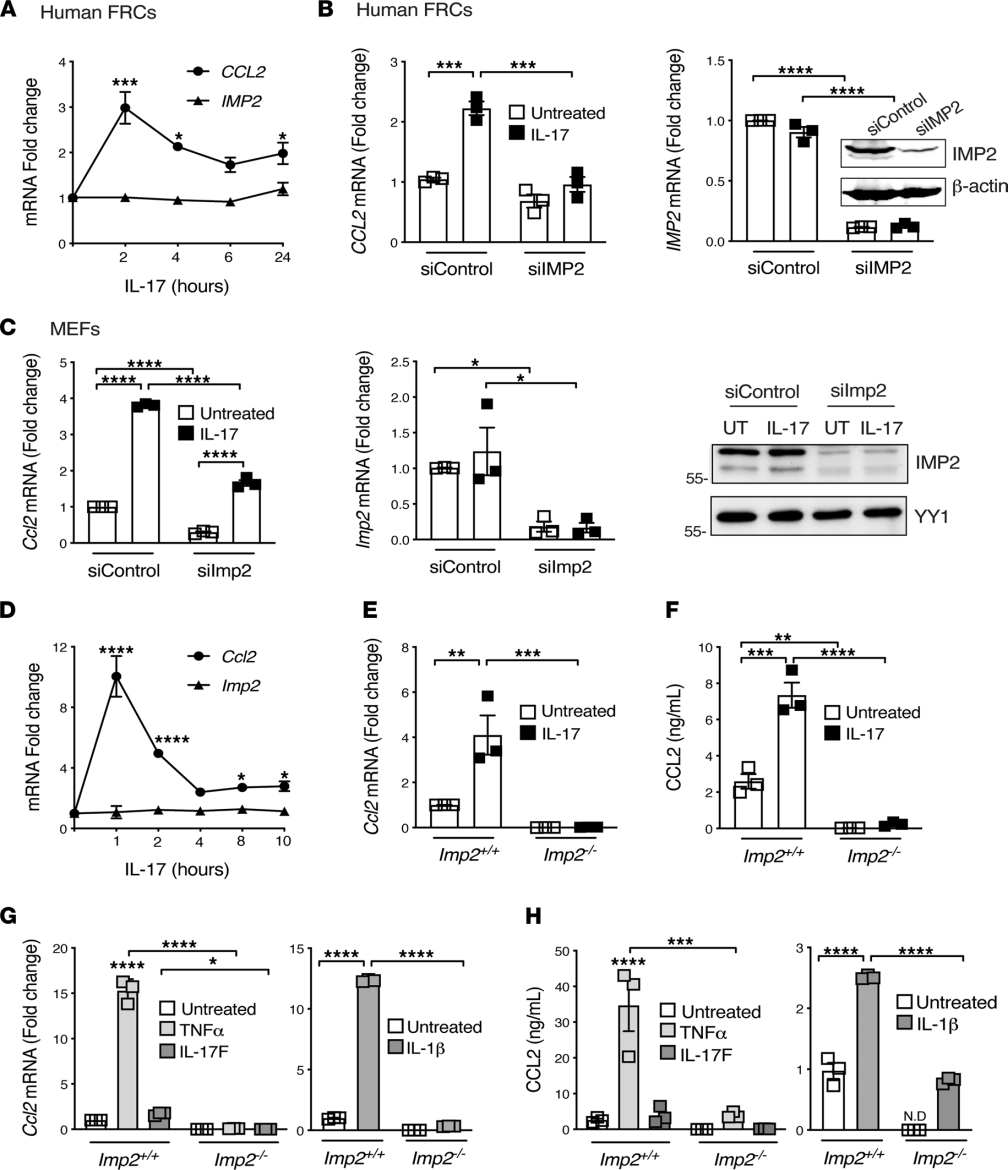
IL-17 induces CCL2 via the RBP IMP2. (**A**) Human tonsillar FRCs were given human IL-17A for the indicated times, and *CCL2* and *IMP2 (IGF2BP2)* were assessed by qPCR. Data shown as mean fold change ± SEM from 3 independent experiments. (**B**) Human FRCs were transfected with siRNAs targeting *IMP2* or scrambled control, treated ± IL-17A for 2 hours and *CCL2* assessed by qPCR. Data are normalized to untreated samples with control siRNA, from 3 independent experiments ± SEM. *Inset:* IMP2 expression assessed by Western blot in human FRCs, representative of 2 independent experiments. (**C**) Primary MEFs were transfected with siRNAs targeting *Imp2* or control and treated ± IL-17A for 8 hours. *Left:* indicated mRNAs were assessed by qPCR. Data are normalized to untreated samples transfected with control siRNA from 3 independent experiments ± SEM. *Right*: IMP2 levels assessed by Western blot in MEFs. (**D**) MEFs were treated with IL-17A and *Ccl2* and *Imp2* were assessed by qPCR normalized to *Gapdh*. Data shown as mean fold change ± SEM from 3 independent experiments. (**E**) *Imp2^+/+^* or *Imp2*^–/–^ MEFs were treated ± IL-17A for 8 hours and *Ccl2* assessed by qPCR. Data shown as mean fold change ± SEM from 3 independent experiments. (**F**) *Imp2^+/+^* or *Imp2*^–/–^ cells were treated with IL-17A for 24 hours, and CCL2 in conditioned supernatants was assessed by ELISA. Data show ng/mL ± SEM from 3 independent experiments. (**G**) *Imp2^+/+^* or *Imp2*^–/–^ MEFs were treated ± IL-17F, TNF-α, IL-1β for 8 hours and *Ccl2* assessed by qPCR. Data shown as mean fold change ± SEM from 3 independent experiments. (**H**) *Imp2^+/+^* or *Imp2*^–/–^ cells were treated ± IL-17F, TNF-α, IL-1β for 24 hours, and CCL2 in conditioned supernatants was assessed by ELISA. Data shown as ng/mL ± SEM from 3 independent experiments. *P < 0.05, **P < 0.01, ***P < 0.001, ****P < 0.0001 by ANOVA with post hoc Tukey’s test.

We next interrogated the role of IMP2 on IL-17 induction of CCL2 in human LN stromal cells, focusing on fibroblastic reticular cells (FRCs) as these are key responders to IL-17. Primary human FRCs (CD45^–^CD31^–^gp38^+^) were treated with IL-17 in the presence or absence of *IMP2* siRNA. IL-17 treatment upregulated *CCL2* in FRCs, peaking at 2 hours and sustained over 24 hours (Figure 5A). Knockdown of IMP2 efficiently abrogated IL-17–mediated upregulation of *CCL2* (Figure 5B). Similarly, *Imp2* silencing suppressed IL-17–mediated upregulation of *Ccl2* in primary murine fibroblasts (Figure 5C), and IL-17–enhanced *Ccl2* mRNA and CCL2 protein were abrogated in *Imp2*^–/–^ MEFs (Figure 5, D–F). IMP2 also regulated *Ccl2* mRNA and protein in response to TNF-α, IL-17F, and IL-1β (Figure 5, G and H). Thus, IMP2 is required for cytokine-induced upregulation of CCL2 in primary mouse and human fibroblasts.

**Figure 6:**
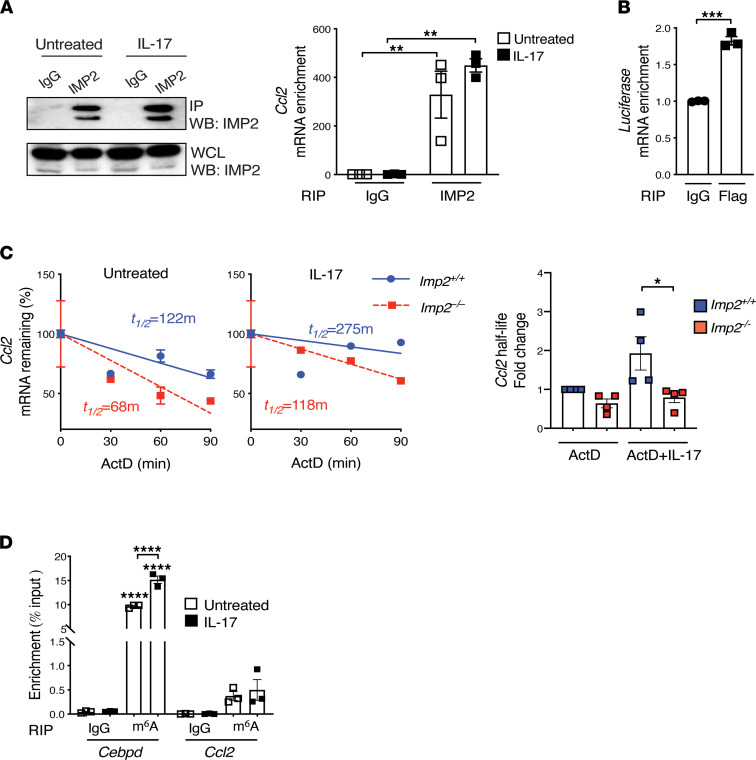
IMP2 binds to Ccl2 mRNA and promotes transcript stability. (**A**) *Imp2^+/+^* MEFs were treated ± IL-17 for 3 hours and subjected to RIP with anti-IMP2 or IgG control Abs. *Left:* IgG and IMP2 immunoprecipitates from *Imp2^+/+^* MEFs, assessed by Western blot. *Right: Ccl2* was assessed by qPCR and normalized to input. Data show mean ± SEM of 3 independent experiments. (**B**) HEK293T cells were cotransfected with IMP2-FLAG, and a Luc reporter was fused to WT *Ccl2*-3′UTR. Lysates were subjected to RIP with anti-FLAG Abs, and Luc mRNA was assessed by qPCR. Data are normalized to input and are representative of 2 independent experiments. (**C**) MEFs were pretreated with TNF-α for 3 hours, then incubated with actinomycin D ± IL-17 for the indicated times, with *Ccl2* assessed by qPCR. Data normalized to time = 0 (designated 100%) and half-life determined by linear regression, as described (26). *Left:* representative data. *Right:* pooled data from 4 independent experiments. (**D**) MEFs were treated ± IL-17 for 3 hours and subjected to RIP with m^6^A or IgG control Abs. qPCR of the indicated mRNAs is presented as percentage input. Data are representative of 2 independent experiments. *P < 0.05, **P < 0.01, ***P < 0.001, ****P < 0.0001 by ANOVA with post hoc Tukey’s test (**A**, **C**, and **D**), unpaired Student’s *t* test (**B**).

IMP2 is known to regulate target mRNA stability and translation, typically by binding to regulatory elements in 3′ or 5′ UTR motifs (22–24). Hence, we evaluated the capacity of IMP2 to bind to *Ccl2* mRNA by RNA IP (RIP). Cells were treated with IL-17, then immunoprecipitated with anti-IMP2 Abs or control IgG, and lysates were evaluated for *Ccl2* mRNA. IMP2 RIP samples were substantially enriched in *Ccl2* mRNA compared with IgG controls, demonstrating that *Ccl2* is a direct IMP2 “client” transcript (Figure 6A). To determine whether IMP2 interacts with the *Ccl2* 3′ UTR, we coexpressed a *Ccl2*-3′ UTR-luciferase fusion construct with FLAG-tagged IMP2 in HEK293 cells. Lysates were subjected to RIP with anti-FLAG Abs, and *Luc* mRNA enrichment assessed by quantitative PCR (qPCR). As shown, there was substantial association of IMP2 with *Luc* mRNA, indicating that IMP2 can bind the *Ccl2* 3′ UTR (Figure 6B). To determine whether IMP2 enhances *Ccl2* RNA stability, *Ccl2* mRNA half-life (*t_½_*) was assessed using a standard actinomycin mRNA decay assay in MEFs (25–27). As shown, IL-17 promoted *Ccl2* longevity approximately 2-fold, increasing the *t_½_* from 122 to 275 minutes (Figure 6C). In both untreated and IL-17–treated conditions, *Ccl2* mRNA had a shorter *t_½_* in *Imp2*^–/–^ compared with *Imp2^+/+^* cells (Figure 6C). Collectively, these data demonstrated that *Ccl2* is an IMP2 client mRNA and that IL-17 enhances *Ccl2* mRNA by promoting transcript stabilization.

We and others recently showed that IMP2 recognizes many of its client mRNAs through the N6-methyladenosine (m^6^A) “epitranscriptomic” RNA modification, for example, *Cebpd* and *Myc* (18, 28, 29). However, *Ccl2* has very few predicted m^6^A consensus sites based on public databases (RMBase and SRAMP). To determine empirically whether *Ccl2* contains m^6^A modifications, we performed RIP with anti-m^6^A Abs. As previously shown, *Cebpd* mRNA was substantially increased upon m^6^A RIP (MeRIP), both at baseline and after IL-17 treatment (18) (Figure 6D). *Ccl2* was also detected in MeRIP samples but at a much lower level than *Cebpd*, which is commensurate with its limited number of predicted m^6^A motifs (Figure 6D).

The RBP Hu-antigen R (HuR) has been implicated in regulating inflammatory mRNAs, including chemokines activated by IL-17 (26, 30). HuR also interacts with multiple binding partners, including IMP2, which determine its target transcript specificity (28, 31). Like IMP2, knockdown of *HuR* impaired expression of *Ccl2* mRNA, in agreement with prior reports (32) (Supplemental Figure 5A). In addition, knockdown of *HuR* in combination with *Imp2* reduced the expression of *Ccl2* more profoundly than knockdown of *Imp2* alone, consistent with a model in which IMP2 and HuR act cooperatively to induce *Ccl2* expression. To determine whether the interaction between IMP2 and HuR is RNA dependent, MEF lysates were treated with RNase A and immunoprecipitated with anti-IMP2 Abs. Although RNAse A treatment had some nonspecific effects on total IMP2 levels, interactions between IMP2 and HuR were reduced (Supplemental Figure 5B), suggesting that RNA bridges or otherwise stabilizes HuR and IMP2 interactions.

### Imp2 deletion after EAE alleviates disease.

**Figure 7:**
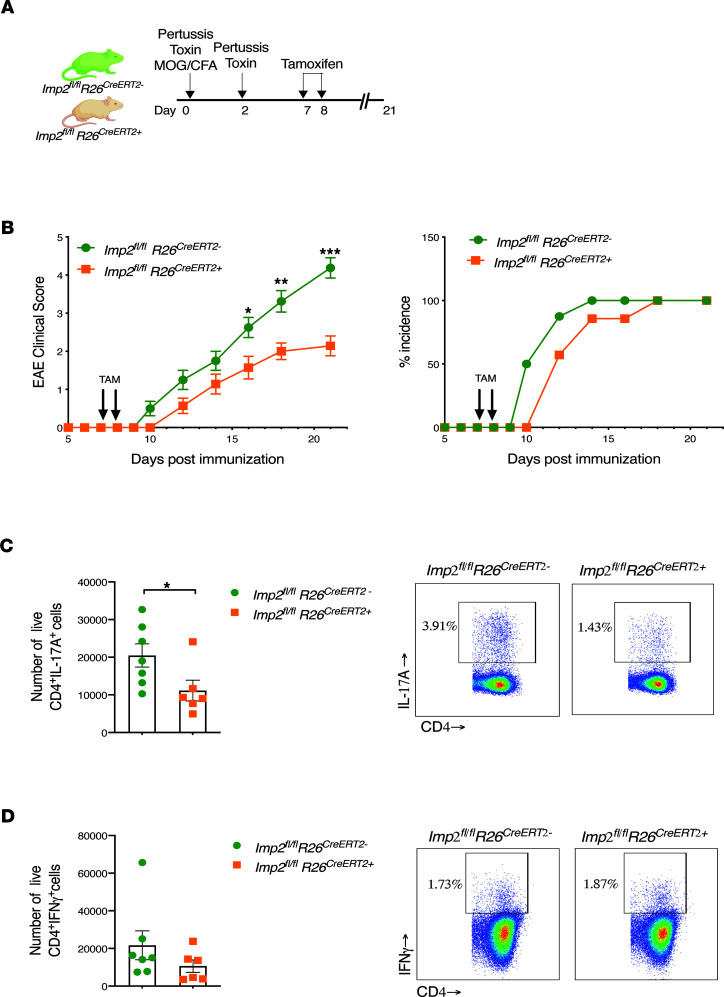
Imp2 deletion after EAE induction decreases EAE. (**A**–**D**) *Imp2^fl/fl^* and *Imp2^fl/fl^ R26^CreERT2^* mice were administered 2 mg tamoxifen (TAM) i.p. for 2 days, starting day 7 after EAE. (**B**) The indicated mice (*Imp2^fl/fl^ R26*^CreERT2–^ *n* = 8 and *Imp2^fl/fl^ R26^CreERT+^ n* = 7) were subjected to EAE and clinical scores assessed daily. *Left:* data are presented as mean clinical score, pooled from 2 independent experiments. *Right*: percentage of mice exhibiting EAE symptoms (incidence). (**C** and **D**) *Imp2^fl/fl^ R26*^CreERT2–^ *n* = 7 and *Imp2^fl/fl^ R26^CreERT+^ n* = 6 mice were subjected to EAE and treated with TAM on days 7 and 8. Lymph nodes were harvested on day 12 and cells treated with PMA/ionomycin for 4 hours. Cells were stained for CD4, IL-17A, and IFN-γ and quantified by flow cytometry. Data were pooled from 2 independent experiments. *Right*: representative FACS plots. Each symbol represents 1 mouse. *P < 0.05, **P < 0.01, ***P < 0.001 by Mann-Whitney test (**B**) or unpaired Student’s *t* test (**C** and **D**).

To date, there are no available pharmacological or other clinically applicable inhibitors of IMP2. To determine whether loss of IMP2 after disease initiation could ameliorate EAE, *Imp2^fl/fl^* mice were crossed to mice with a constitutive tamoxifen-inducible Cre (*Rosa26^CreERT2^*), thus permitting gene deletion only upon administration of tamoxifen. Mice were given tamoxifen at days 7 and 8 after induction of EAE, and disease signs were scored through day 21 (Figure 7A). Mice in which IMP2 was deleted after MOG immunization (*Imp2^fl/fl^ Rosa26^CreERT2+^*) showed reduced EAE clinical signs as well as delayed disease incidence (Figure 7B). Consistent with this, mice also exhibited reduced numbers of Th17 cells but not Th1 cells in draining LNs (Figure 7, C and D). These data showed that the effects of IMP2 on inflammation are not developmental and that blockade of IMP2 could be the basis of a therapeutic approach in autoimmune disease.

## Discussion

CCL2 is best recognized for its ability to promote monocyte recruitment during host defense (33). CCL2 also drives pathogenic inflammation and has therefore been implicated in many autoinflammatory diseases, including multiple sclerosis (3–6). Hence, tight regulation of CCL2 is crucial to maintain a balanced immune response that permits immunity to infection without causing autoimmune sequelae or a cytokine storm.

Here, we showed that amplification of *Ccl2* is mediated by IL-17 in mesenchymal cells from mouse as well as human LN stroma. Often, induction of inflammatory genes is viewed in the context of promoter regulation, but many immune genes, including *Ccl2*, are expressed at baseline tonic levels, and their capacity to be induced during immune insult is due to expanded transcript half-life or other posttranscriptional mechanisms (34). IMP2, like its binding partner HuR, promotes *Ccl2* transcript stabilization, which is ultimately required for enhanced CCL2 secretion from LN stromal cells (32, 35). In addition, IMP2 and HuR interact in an RNA-dependent manner to stabilize *Ccl2* mRNA. While IMP2 functions primarily in nonhematopoietic cells by virtue of selective expression in these cell types (18), HuR is more widely expressed and has more profound impacts on Th17 cells in addition to acting on IL-17–responsive cells (30, 36, 37). Therefore, it is likely that HuR also drives IMP2-independent roles in disease pathogenesis. Given the prominent role of CCL2 in inflammation, there are also negative regulators of *Ccl2* mRNA that offset stabilizing effects. For example, tristetraprolin (TTP, *Zfp36*) is a well-characterized zinc finger RBP that destabilizes *Ccl2* mRNA (35). Hence, multiple RBPs converge on *Ccl2* expression control.

Most of what is known about IMP2 comes from studies of metabolism and cancer (17). As suggested by its gene name *Igf2bp2* (IGF2 mRNA-binding protein 2), IMP2 promotes expression of IGF2 by binding to its 5′ UTR and controlling translation (38). In this regard, altered expression of components of the IGF system have been observed in multiple sclerosis (39–41). However, in contrast to IMP2, IGF2 alleviates EAE pathogenesis, and mice treated with IGF2-neutralizing antibodies showed increased EAE disease severity (42).

Mice lacking IMP2 are less susceptible to AGN driven by IL-17–dependent regulation of C/EBP transcription factors, specifically in the nonhematopoietic compartment (18). Although C/EBPs are implicated in CCL2 regulation, their expression in the LNs and spinal cords of *Imp2*^–/–^ mice is not altered. Indeed, C/EBP expression in the myeloid cell lineage is implicated in EAE pathogenesis (43), rather than its expression in the stromal compartment where IMP2 is mostly expressed. We and others recently showed that IMP2 recognizes many of its client mRNAs through m^6^A RNA modification (18, 28). However, according to public databases, *Ccl2* has only a few predicted m^6^A consensus sites within its 3′ UTR (44, 45), and results from our m^6^A RIP pulldowns showed only modest enrichment of *Ccl2* mRNA. Interestingly, a recent report indicates that *Ccl2* mRNA stability can also be negatively regulated by m^6^A through binding of the destabilizing protein TTP and the m^6^A reader YTHDF2. Moreover, TTP promoted global increases in m^6^A methylation through upregulation of the methylation machinery (46). Therefore, the impact of this RNA modification can be both positive and negative depending on context.

Although *Ccl2* was upregulated in the CNS of *Imp2*^+/+^ mice during EAE, its expression was not statistically reduced in the *Imp2*^–/–^ CNS. This result suggests that IMP2 may exert distinct effects in different tissues. Moreover, IMP2 is enriched in the CNS and is required for axon pathfinding and neurogenesis after hypoxic/ischemic brain injury (47–49). Even so, since loss of IMP2 after onset of EAE alleviates disease, it is unlikely that these developmental activities are needed for autoimmune pathogenesis. Ultimately, tissue-specific deletions of IMP2 will be needed to dissect its roles in given cell types relevant to EAE.

The vital role of fibroblasts in controlling inflammation during autoimmunity has become increasingly appreciated (50, 51). Our finding that LN stromal cells express high levels of CCL2 agrees with work showing that nonhematopoietic stromal cells in LN, especially FRCs, serve as the major source of CCL2 after immunization-induced inflammation (9). Like CCL2, IMP2 is expressed almost exclusively in nonhematopoietic cells, which is in concordance with its role in regulating *Ccl2* expression. Similarly, IL-17 signaling occurs predominantly in nonhematopoietic cell types (13). Moreover, in EAE, IL-17 drives metabolic reprogramming to support proliferation of FRCs during LN expansion, as well as enhancing expression of inflammatory cytokines (20).

Beyond their well-established role in providing structural support to organs, stromal and mesenchymal cells are actively involved in immune responses. FRCs in lymphoid organs regulate adaptive immunity, mesenchymal cells in adipose tissue promote Treg cell differentiation, and stromal cells in the synovium are key determinants of the inflammatory response during arthritis (20, 50, 52–54). CCL2 was reported to modulate Th17 cell differentiation directly (8), but we observed no impact of CCL2 on promoting Th17 polarization, regardless of differentiation conditions employed. Although there was no detectable effect of IMP2 on Th17 polarization under standard differentiation conditions, IL-17 production was lower in *Imp2*-deficient T cells when cultured in suboptimal differentiation conditions, although the basis for this remains unknown. Therefore, we posit that IMP2 orchestrates a feed-forward amplification loop that sustains Th17 generation, primarily by stabilizing *Ccl2* mRNA in stromal cells and promoting monocyte recruitment and to a much lesser extent by acting directly on T cells to promote Th17 differentiation.

In summary, we have uncovered a pathway that dictates posttranscriptional regulation of *Ccl2* mRNA occurring within LN stromal cells and mediated by IL-17. The resulting increase in CCL2 leads to enhanced monocyte recruitment and Th17 cell activation, which is the basis for inflammatory pathogenesis mediated by IL-17. Therapeutic IL-17 blockade is highly effective in psoriasis and psoriatic arthritis and is being explored in other conditions (19). Preliminary trials of anti–IL-17A biologics in multiple sclerosis have shown some promising hints of efficacy (55, 56), although this cytokine is certainly not the sole factor driving pathogenesis (57). Strikingly, deletion of IMP2 after EAE onset ameliorated signs of disease, suggesting that IMP2 could be a viable pharmacological target. RNA-based therapeutic approaches are gaining momentum, as they can potentially be highly specific and act on conventionally undruggable targets (58). Hence, understanding the molecular players involved in RNA regulation may ultimately offer new therapeutic avenues.

## Methods

### Study design.

The objective of this study was to determine how CCL2 is regulated in response to IL-17. We used cell culture studies and an EAE mouse model to define the role of IMP2 in IL-17–induced CCL2 production. Sample sizes were determined by power analyses from pilot or previously published data. Experiments were done 2–3 independent times. Data from multiple experiments were pooled unless noted.

### Mice.

All mice were on the C57BL/6 background. Cohorts were age 8–12 weeks and sex matched. *Imp2*^–/–^ mice were from Oxford University, Oxford, United Kingdom. WT controls were generated in house by breeding. *Imp2^fl/f^* and *Rosa26*^ERT2^ mice were from The Jackson Laboratory (stock number 008463). *Il17ra*^–/–^ mice were provided by Amgen. Mice were housed in specific pathogen–free conditions. Protocols were approved by the University of Pittsburgh IACUC.

### EAE.

Mice were immunized in 2 sites on the back (s.c.) with 100 μg MOG peptide (35-55; Biosynthesis) emulsified with CFA with M. tuberculosis strain H37Ra (DIFCO Laboratories). Mice also received 100 ng (i.p.) pertussis toxin (List Biological Laboratories) on day 0 and day 2. Mice were assessed daily and scored as follows: 1, flaccid tail; 2, impaired righting reflex and hindlimb weakness; 3, partial hindlimb paralysis; 4, complete hindlimb paralysis; 5, hindlimb paralysis with partial forelimb paralysis; 6, moribund. For *Imp2*-inducible deletion, mice were administered tamoxifen (2 mg, i.p.) on days 7 and 8 after EAE induction.

### Cell culture and cytokine stimulation.

MEF cells (made from C57BL/6J or *Imp2*^–/–^ mice, both male and female) were cultured in α-MEM (Sigma-Aldrich) with l-glutamine, antibiotics, and 15% FBS. Human stromal cells were cultured in RPMI (α-MEM; Sigma-Aldrich), with l-glutamine, antibiotics, and 10% FBS. IL-17A and IL-17F (PeproTech) were used at 200 ng/mL. TNF-α and IL-1β (PeproTech) were used at 10 ng/mL. Actinomycin D (Sigma-Aldrich) was used at 10 μg/mL.

### Human FRCs.

Human tonsils from patients with sleep apnea were incubated in digestion medium (RPMI, 0.1 mg/mL; DNase I, Invitrogen; and 0.2 mg/mL Liberase, Roche). Single-cell suspensions were filtered and subjected to red blood cell lysis. CD45^+^CD31^+^ cells were magnetically removed using an EasySep Human APC Positive Selection kit (StemCell Technologies). The gp38^+^ FRC population was positively selected using an EasySep Human PE Positive Selection kit (StemCell Technologies) and rested for 2–3 days. Purity of the FRC populations was tested by flow cytometry and was greater than 95%.

### siRNA transfection.

ON-TARGETplus SMARTpool siRNAs targeting IMP2 were from Dharmacon. For RNA silencing, MEFs and human stromal cells were seeded overnight in antibiotic-free media. Transfection was performed 18 hours later with 50 nM siRNA in DharmaFECT reagent 1. Culture media was replaced after 24 hours, and cytokines were administered 24 hours later.

### qPCR, RNA-Seq, and RIP.

RNA was isolated with RNeasy Mini Kits (QIAGEN). cDNA was synthesized by SuperScript III First Strand Kits (Thermo Fisher Scientific). Real-time qPCR was performed with SYBR Green FastMix ROX (Quanta Biosciences) on a 7300 Real-Time instrument (Applied Biosystems) normalized to *Gapdh*. Primers were from QuantiTect Primer Assays (QIAGEN).

For RNA-Seq, cDNA libraries were prepared from LN RNA harvested on day 7 after EAE induction (Nextera XT Kit), and RNA-Seq was performed on the Illumina NextSeq 500 platform by the Health Sciences Sequencing Core at the University of Pittsburgh. Sequencing reads were annotated and aligned to the University of California, Santa Cruz mouse reference genome (mm10, GRCm38.75) using HISAT. HISAT alignment files were used to generate read counts for each gene, and determination of differential gene expression was performed using the DESeq package from Bioconductor. Unbiased hierarchical clustering of differentially expressed genes with *P* less than 0.05 was calculated using CLC Genomics Workbench. Relative expression values in heatmaps are reads per kilobase million values per sample that have been divided by the average expression across all samples.

For IMP2 RIPs, extracts were isolated with lysis buffer (100 mM KCl, 5 mM MgCl_2_, 10 mM HEPES [pH 7.0], 0.5% NP-40, 1 mM dithiothreitol) with RNAse Out (100 U/mL, Invitrogen). Buffers included a protease inhibitor cocktail (Sigma-Aldrich). Lysates were precleared protein A agarose (Roche Applied Science) and immunoprecipitated with Abs against IMP2 (MBL; RN008P) or isotype control (MBL; PM035). Beads were washed with NT2 buffer and digested with DNase I (Roche Applied Science) and protease K (Sigma-Aldrich). Total RNA was extracted with acid phenol.

For MeRIP, 20 to 50 μg of RNA was fragmented and purified by ethanol precipitation. A total of 0.1 fmol of a control m^6^A-modified Gaussia Luc RNA or unmodified Cypridina Luc RNA (supplied with the EpiMark N6-Methyladenosine Enrichment Kit; New England Biolabs; E1610S) were spiked in each sample. For RIP, Protein G Dynabeads (Thermo Fisher Scientific) were washed in MeRIP buffer (150 mM NaCl, 10 mM Tris-HCl [pH 7.5], and 0.1% NP-40) and incubated with anti-m^6^A Abs for 2 hours at 4°C. After washing, anti-m^6^A conjugated beads were incubated with mRNA for 4 hours in RNasin (Promega). Up to 3% of mRNA was used for input. Beads were washed with MeRIP buffer, low-salt wash buffer (50 mM NaCl, 10 mM Tris-HCl [pH 7.5], and 0.1% NP-40), and wash buffer (500 mM NaCl, 10 mM Tris-HCl [pH 7.5], and 0.1% NP-40). m^6^A-modified RNA was eluted in MeRIP buffer containing 5 mM m^6^A salt (Santa Cruz Biotechnology). Eluates were pooled and concentrated by ethanol precipitation. Input and IP fractions were reverse-transcribed using the iScript cDNA synthesis kit (Bio-Rad) and subjected to qPCR.

### RNA decay analysis.

MEFs were primed with TNF-α (10 ng/mL) for 3 hours. Cells were washed with PBS and treated with 10 μg/mL actinomycin D (Sigma-Aldrich) ± 200 ng/mL IL-17 as described (26). Levels of mRNA were assessed by qPCR. For each mRNA, remaining quantity (%) was calculated by normalizing ΔΔCt to the ΔΔCt of samples primed with TNF-α.

### In vitro T cell differentiation.

Naive splenic CD4^+^ T cells were purified by magnetic separation (Miltenyi Biotec). T cells were activated with anti-CD28 (5 mg/mL; Bio X Cell clone PV-1, catalog BE0015-5) and plate-bound anti-CD3 (clone 145-TC11, 5 mg/mL; Bio X Cell). Cells were either treated with full RPMI for Th0 conditions or with RPMI containing optimal Th17 conditions (IL-1β, 40 ng/mL; IL-6, 100 ng/mL; IL-23, 20 ng/mL; and TGF-β, 10 ng/mL; with or without CCL2, 5 ng/mL) or Th1 (IL-12, 10 ng/mL) for 3 days. Experiments were also performed in suboptimal Th17 conditions (TGF-β, 5 ng/mL and IL-6, 20 ng/mL, with or without CCL2, 5 ng/mL). Cytokines were from R&D Systems.

### Flow cytometry.

CNS tissue homogenates were incubated in digestion medium containing 0.5 mg/mL collagenase type I (Worthington Biochemical) and 1000 U/mL DNase I (Sigma-Aldrich) for 45 minutes following myelin debris removal using a Percoll gradient. LNs were incubated in digestion medium RPMI supplemented with 0.1 mg/mL DNase I (Invitrogen) and 0.1 mg/mL Liberase (Roche). Collected single-cell suspensions were filtered. Resulting cell suspensions were incubated with the following antibodies: anti-CD45 (clone 30-F11, Thermo Fisher Scientific), anti-Ly6G (clone 1A8, BioLegend), and anti-Ly6C (clone HK1.4, BioLegend). Dead cells were excluded using Ghost Dye (eBioscience). For cytokine analysis, LNs were cultured in complete medium (RPMI media containing 10% FCS, supplemented with l-glutamine and antibiotics) with 50 ng/mL PMA and 500 ng/mL ionomycin (Sigma-Aldrich) in the presence of GolgiPlug (BD Biosciences) for 4 hours followed by FACS staining. Cells were stained with Ghost Dye, CD4 (clone GK1.5, BioLegend), IL-17 (clone TC11-18H10.1, BioLegend), IL-10 (JES5-16E3, BioLegend), and IFN-γ (XMG1.2, BioLegend). To assess T cell differentiation, in vitro differentiated CD4^+^ T cells were stained with Ghost Dye, CD4 (clone GK1.5, BioLegend), and IL-17 (clone TC11-18H10.1, BioLegend). Samples were acquired with BD LSRFortessa and analyzed using FlowJo software (Tree Star).

### Immune cell isolation.

LNs from naive mice were incubated in digestion medium RPMI supplemented with 0.1 mg/mL DNase I (Invitrogen) and 0.1 mg/mL Liberase (Roche). Single-cell suspensions were incubated with CD45 and anti–Ter-119 microbeads (Miltenyi Biotec). After selection (MACS column, Miltenyi Biotec), CD45^–^ and CD45^+^ populations were plated overnight in complete RPMI. Cells were stimulated with IL-17 (200 ng/mL) for 24 hours.

### Immunoblotting and ELISA.

The following immunoblotting Abs were used: mouse IMP2 (MBL, 1:1000; RN008P), human IMP2 (Cell Signaling Technology, 1:1000; 14672), YY1 (Santa Cruz Biotechnology, 1:1000; sc-1703), and β-actin (Abcam, 1:25,000; ab49900). Western blots were developed with a FluorChem E imager (Protein Simple). CCL2, IL-17, and IFN-γ ELISA kits were used per the manufacturer’s instructions (R&D Systems).

### Data availability.

*Imp2*^–/–^ mice are available by material transfer agreement (MTA) from Oxford University. *Il17ra*^–/–^ mice are under MTA from Amgen. All other mice are commercially available. RNA-Seq data are available through the National Center for Biotechnology Information’s Gene Expression Omnibus under accession number GSE180561.

### Statistics.

One-way ANOVA with post hoc Tukey’s analysis or Mann-Whitney test or 2-tailed Student’s *t* test were used to assess statistical significance, with *P* less than 0.05 considered significant. Data were analyzed with GraphPad Prism. Each symbol represents 1 mouse unless indicated. *P* values less than 0.05 were considered significant.

### Study approval.

All the experiments were conducted following NIH guidelines under protocols approved by the University of Pittsburgh IACUC (protocol 20037105).

## Author contributions

RB, NA, SLG, and MJM conceptualized the study; RB, NA, CZ, QL, and SM devised the methodology; RB, NA, CZ, QL, and SM conducted the investigation; RB and SLG wrote the original draft; NA, MJM, CZ, and QL reviewed and edited the manuscript; SLG and MJM acquired funding; SLG and MJM supervised the study.

## Supplementary Material

Supplemental data

## Version 1 12/16/2021

In-Press Preview

## Version 2 02/08/2022

Electronic publication

## References

[1] Ransohoff RM (2012). Animal models of multiple sclerosis: the good, the bad and the bottom line. Nat Neurosci.

[2] Karpus WJ (1997). Differential CC chemokine-induced enhancement of T helper cell cytokine production. J Immunol.

[3] Kennedy KJ (1998). Acute and relapsing experimental autoimmune encephalomyelitis are regulated by differential expression of the CC chemokines macrophage inflammatory protein-1alpha and monocyte chemotactic protein-1. J Neuroimmunol.

[4] Fife BT (2000). CC chemokine receptor 2 is critical for induction of experimental autoimmune encephalomyelitis. J Exp Med.

[5] Izikson L (2000). Resistance to experimental autoimmune encephalomyelitis in mice lacking the CC chemokine receptor (CCR)2. J Exp Med.

[6] Huang DR (2001). Absence of monocyte chemoattractant protein 1 in mice leads to decreased local macrophage recruitment and antigen-specific T helper cell type 1 immune response in experimental autoimmune encephalomyelitis. J Exp Med.

[7] Kara EE (2015). CCR2 defines in vivo development and homing of IL-23-driven GM-CSF-producing Th17 cells. Nat Commun.

[8] Bakos E (2017). CCR2 regulates the immune response by modulating the interconversion and function of effector and regulatory T cells. J Immunol.

[9] Dasoveanu DC (2020). Lymph node stromal CCL2 limits antibody responses. Sci Immunol.

[10] Martin EW (2020). Integrative analysis suggests cell type-specific decoding of NF-κB dynamics. Sci Signal.

[11] Wojkowska DW (2017). Interleukin 17A promotes lymphocytes adhesion and induces CCL2 and CXCL1 release from brain endothelial cells. Int J Mol Sci.

[12] Kebir H (2007). Human TH17 lymphocytes promote blood-brain barrier disruption and central nervous system inflammation. Nat Med.

[13] Li X (2019). IL-17 receptor-based signaling and implications for disease. Nat Immunol.

[14] Hildebrand DG (2013). IκBζ is a transcriptional key regulator of CCL2/MCP-1. J Immunol.

[15] Carpenter S (2014). Post-transcriptional regulation of gene expression in innate immunity. Nat Rev Immunol.

[16] Degrauwe N (2016). IMPs: an RNA-binding protein family that provides a link between stem cell maintenance in normal development and cancer. Genes Dev.

[17] Dai N (2020). The diverse functions of IMP2/IGF2BP2 in metabolism. Trends Endocrinol Metab.

[18] Bechara R (2021). The m^6^A reader IMP2 directs autoimmune inflammation through an IL-17- and TNFα-dependent C/EBP transcription factor axis. Sci Immunol.

[19] McGeachy MJ (2019). The IL-17 family of cytokines in health and disease. Immunity.

[20] Majumder S (2019). IL-17 metabolically reprograms activated fibroblastic reticular cells for proliferation and survival. Nat Immunol.

[21] Majumder S, McGeachy MJ (2021). IL-17 in the pathogenesis of disease: good intentions gone awry. Annu Rev Immunol.

[22] Dai N (2015). IGF2BP2/IMP2-deficient mice resist obesity through enhanced translation of Ucp1 mRNA and other mRNAs encoding mitochondrial proteins. Cell Metab.

[23] Conway AE (2016). Enhanced CLIP uncovers IMP protein-RNA targets in human pluripotent stem cells important for cell adhesion and survival. Cell Rep.

[24] Degrauwe N (2016). The RNA binding protein IMP2 preserves glioblastoma stem cells by preventing let-7 target gene silencing. Cell Rep.

[25] Henness S (2004). IL-17A augments TNF-alpha-induced IL-6 expression in airway smooth muscle by enhancing mRNA stability. J Allergy Clin Immunol.

[26] Amatya N EE C (2018). IL-17 integrates multiple self-reinforcing, feed-forward mechanisms through the RNA binding protein Arid5a. Sci Signal.

[27] Masuda K (2013). Arid5a controls IL-6 mRNA stability, which contributes to elevation of IL-6 level in vivo. Proc Natl Acad Sci U S A.

[28] Huang H (2018). Recognition of RNA N^6^-methyladenosine by IGF2BP proteins enhances mRNA stability and translation. Nat Cell Biol.

[29] Bechara R, Gaffen SL (2021). ‘(m^6^)A’ stands for ‘autoimmunity’: reading, writing, and erasing RNA modifications during inflammation. Trends Immunol.

[30] Herjan T (2013). HuR is required for IL-17-induced Act1-mediated CXCL1 and CXCL5 mRNA stabilization. J Immunol.

[31] Hafner M (2010). Transcriptome-wide identification of RNA-binding protein and microRNA target sites by PAR-CLIP. Cell.

[32] Fan J (2011). Chemokine transcripts as targets of the RNA-binding protein HuR in human airway epithelium. J Immunol.

[33] Gschwandtner M (2019). More than just attractive: how CCL2 influences myeloid cell behavior beyond chemotaxis. Front Immunol.

[34] Kafasla P (2014). Post-transcriptional coordination of immunological responses by RNA-binding proteins. Nat Immunol.

[35] Panganiban RP (2014). Coordinated post-transcriptional regulation of the chemokine system: messages from CCL2. J Interferon Cytokine Res.

[36] Chen J (2013). Posttranscriptional gene regulation of IL-17 by the RNA-binding protein HuR is required for initiation of experimental autoimmune encephalomyelitis. J Immunol.

[37] Herjan T (2018). IL-17-receptor-associated adaptor Act1 directly stabilizes mRNAs to mediate IL-17 inflammatory signaling. Nat Immunol.

[38] Dai N (2011). mTOR phosphorylates IMP2 to promote IGF2 mRNA translation by internal ribosomal entry. Genes Dev.

[39] Chesik D (2006). Insulin-like growth factor binding proteins: regulation in chronic active plaques in multiple sclerosis and functional analysis of glial cells. Eur J Neurosci.

[40] Lanzillo R (2011). Insulin-like growth factor (IGF)-I and IGF-binding protein-3 serum levels in relapsing-remitting and secondary progressive multiple sclerosis patients. Eur J Neurol.

[41] DiToro D (2020). Insulin-like growth factors are key regulators of T helper 17 regulatory T cell balance in autoimmunity. Immunity.

[42] Du L (2019). IGF-2 preprograms maturing macrophages to acquire oxidative phosphorylation-dependent anti-inflammatory properties. Cell Metab.

[43] Screpanti I (1995). Lymphoproliferative disorder and imbalanced T-helper response in C/EBP beta-deficient mice. EMBO J.

[44] Zhou Y (2016). SRAMP: prediction of mammalian N6-methyladenosine (m6A) sites based on sequence-derived features. Nucleic Acids Res.

[45] Xuan JJ (2018). RMBase v2.0: deciphering the map of RNA modifications from epitranscriptome sequencing data. Nucleic Acids Res.

[46] Xiao P (2021). TTP protects against acute liver failure by regulating CCL2 and CCL5 through m6A RNA methylation. JCI Insight.

[47] Preitner N (2016). IMP2 axonal localization, RNA interactome, and function in the development of axon trajectories. Development.

[48] Zhu X (2019). RBM3 promotes neurogenesis in a niche-dependent manner via IMP2-IGF2 signaling pathway after hypoxic-ischemic brain injury. Nat Commun.

[49] Fujii Y (2013). IMP2 regulates differentiation potentials of mouse neocortical neural precursor cells. Genes Cells.

[50] Krishnamurty AT, Turley SJ (2020). Lymph node stromal cells: cartographers of the immune system. Nat Immunol.

[51] Buechler MB, Turley SJ (2018). A short field guide to fibroblast function in immunity. Semin Immunol.

[52] Nygaard G, Firestein GS (2020). Restoring synovial homeostasis in rheumatoid arthritis by targeting fibroblast-like synoviocytes. Nat Rev Rheumatol.

[53] Spallanzani RG (2019). Distinct immunocyte-promoting and adipocyte-generating stromal components coordinate adipose tissue immune and metabolic tenors. Sci Immunol.

[54] Kohlgruber A (2018). γδ T cells producing interleukin-17A regulate adipose regulatory T cell homeostasis and thermogenesis. Nat Immunol.

[55] Havrdova E (2016). Activity of secukinumab, an anti-IL-17A antibody, on brain lesions in RRMS: results from a randomized, proof-of-concept study. J Neurol.

[56] Volpe E (2015). Advances in T helper 17 cell biology: pathogenic role and potential therapy in multiple sclerosis. Mediators Inflamm.

[57] Lovett-Racke AE (2011). Th1 versus Th17: are T cell cytokines relevant in multiple sclerosis?. Biochim Biophys Acta.

[58] Wang F (2020). RNA therapeutics on the rise. Nat Rev Drug Discov.

