# Supplementary material for: The RNA-binding protein IMP2 drives a stromal-Th17 cell circuit in autoimmune neuroinflammation
Source: JCI Insight. 2022 Feb 8;7(3):e152766. doi: 10.1172/jci.insight.152766 (PMC8855811; doi:10.1172/jci.insight.152766)
Supplement: Supplemental data [file jciinsight-7-152766-s156.pdf]

SUPPLEMENTAL DATA

The RNA binding protein IMP2 drives a stromal-Th17 cell circuit in autoimmune neuroinflammation

Rami Bechara [1,2 ‡], Nilesh Amatya [2], Saikat Majumder [2], Chunsheng Zhou[2], Yang Li[2], Qixing Liu[2,3],

Mandy J. McGeachy [2] and Sarah L. Gaffen [2‡]

[1] Université Paris-Saclay, CEA, INSERM UMR 1184, Centre de Recherche en Immunologie des Infections Virales et des Maladies Auto-Immunes, Le Kremlin Bicêtre, France

[2] Division of Rheumatology & Clinical Immunology, University of Pittsburgh, Pittsburgh, PA, USA

[3] Tsinghua University, Beijing, PR China

Supp Figure 1

A

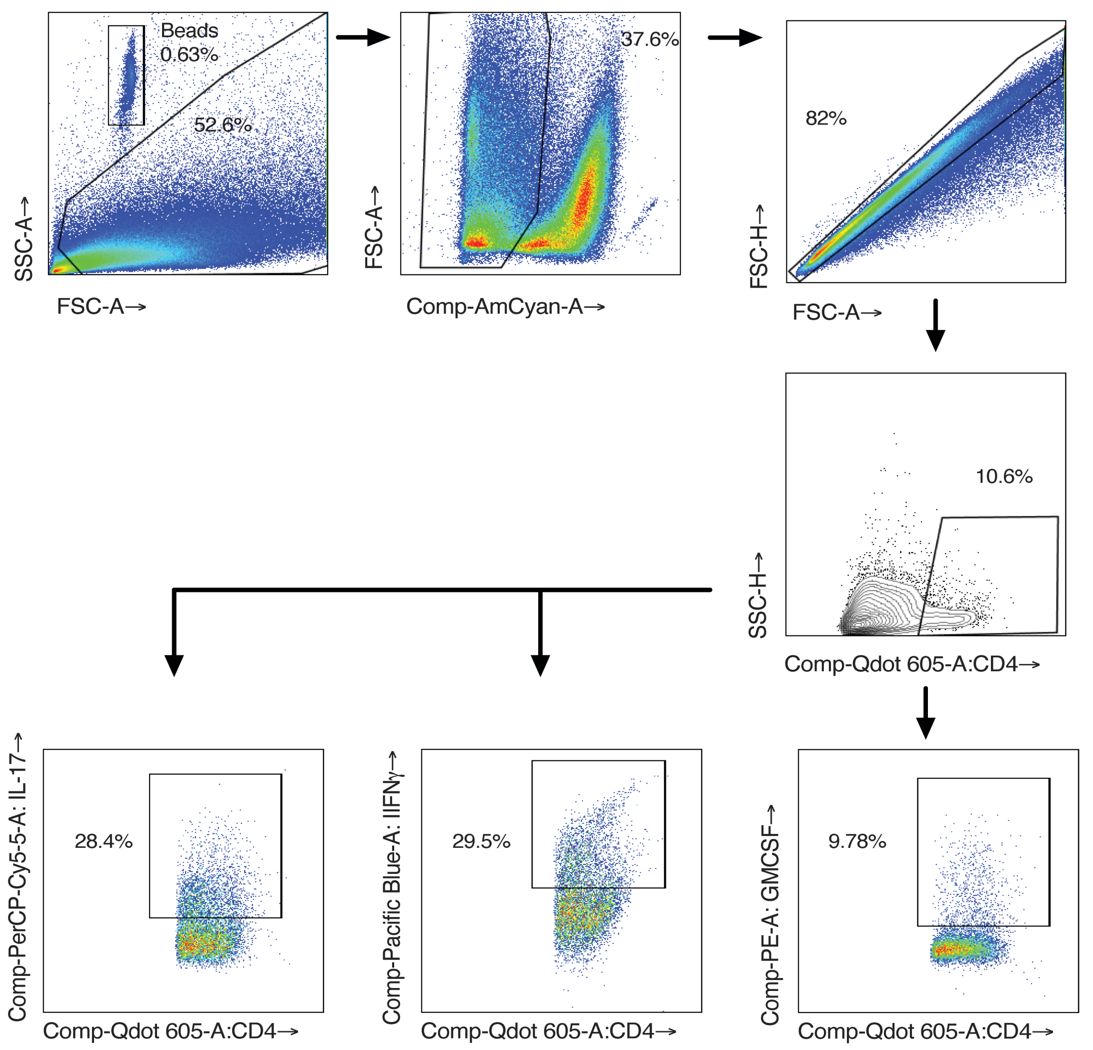

***Supplementary Fig 1. Gating strategy used to characterize cytokines in CNS of EAE Imp2*$^{+/+}$and *Imp2*$^{-/-}$ mice**

Imp2$^{+/+}$and *Imp2*$^{-/-}$ mice were subjected to EAE. CNS homogenates were prepared on day 16 and cells treated with PMA/ionomycin for 4 h. Gating strategy is indicated.

Supp Figure 2

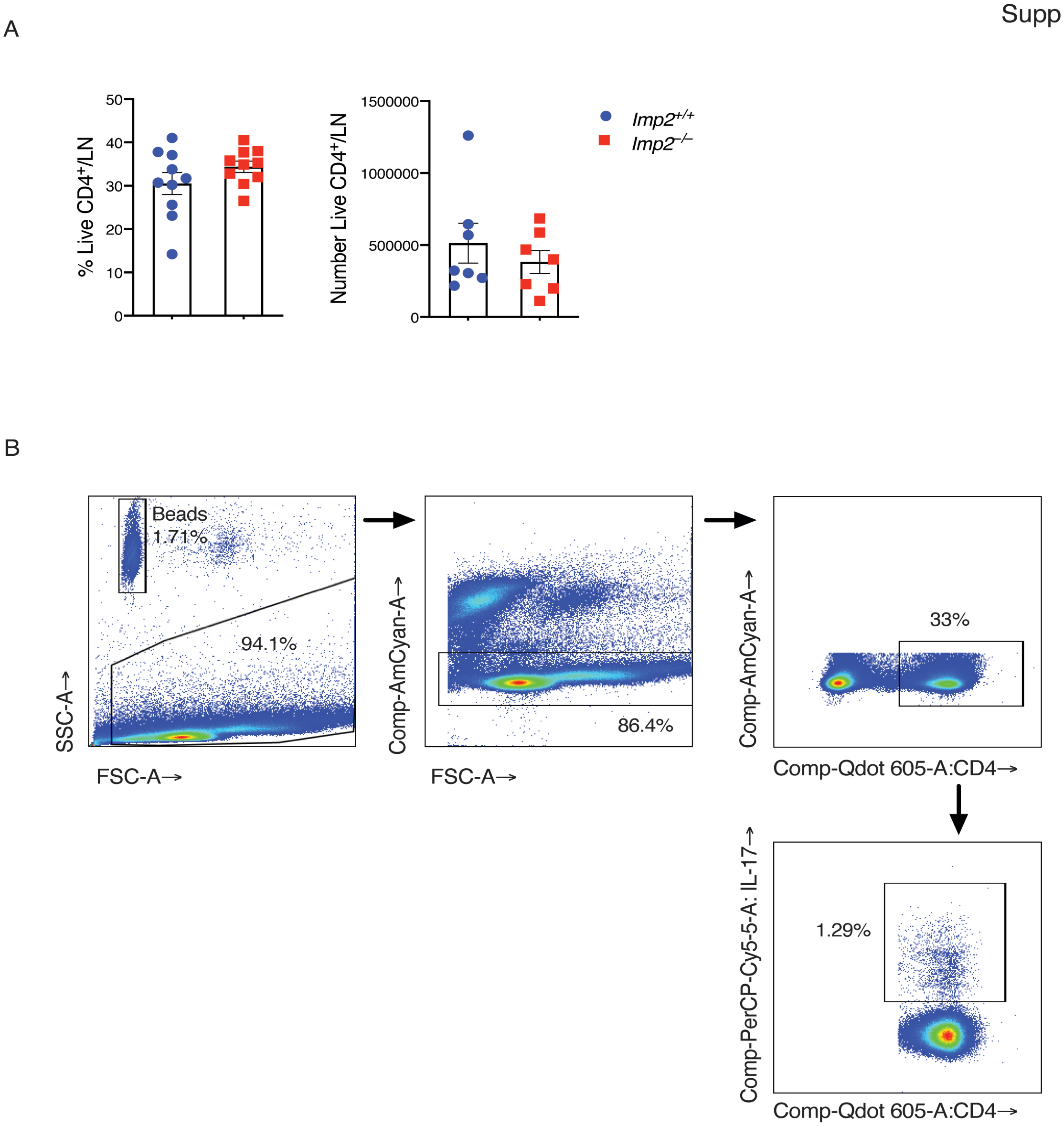

***Supplementary Fig 2. Total CD4$^+$ T cell assessment in LNs of EAE Imp2$^{+/+}$*and *Imp2$^{-/-}$* mice**

*Imp2$^{+/+}$*and *Imp2$^{-/-}$* mice were subjected to EAE. Inguinal LN homogenates were prepared on day 10 and cells treated with PMA/ionomycin for 4 h. (A) Live CD4$^+$ cells were determined by staining for CD4. Graphs show the numbers and percentages of live CD4$^+$ cells, pooled from 2-3 independent experiments. Each symbol represents one mouse. (B) Gating strategy used to characterize cytokines in LNs.

Supp Figure 3

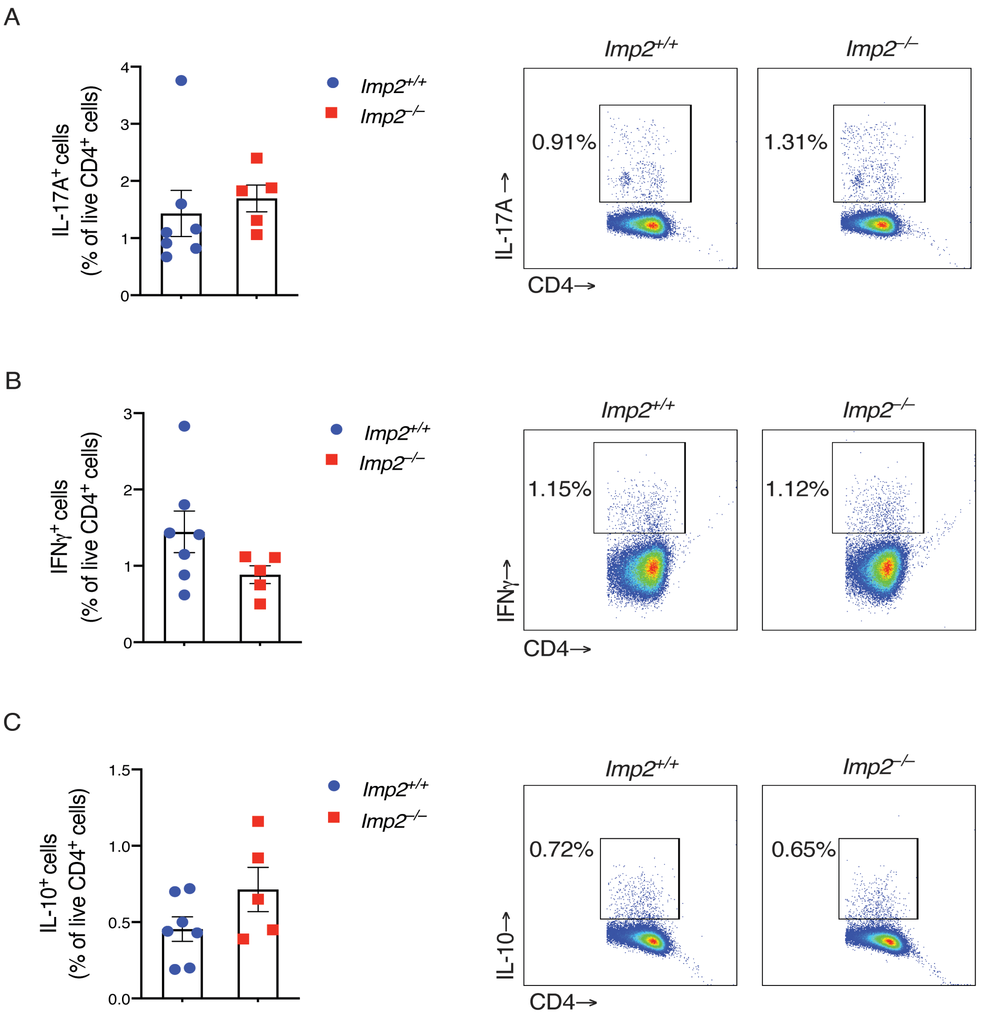

Supplementary Fig 3. T cell cytokine profiles in LNs of Imp2$^{-/-}$ mice

Inguinal LN homogenates were prepared from naïve mice and cells treated with PMA/ionomycin for 4 h. (A-C) *Left:* Cells were stained for CD4, IL-17A, IL-10 and IFNγ, and quantified by flow cytometry. Data were pooled from 2 independent experiments. Each symbol represents one mouse. *Right:* Representative FACS plots from 2 independent experiments.

Supp Figure 4

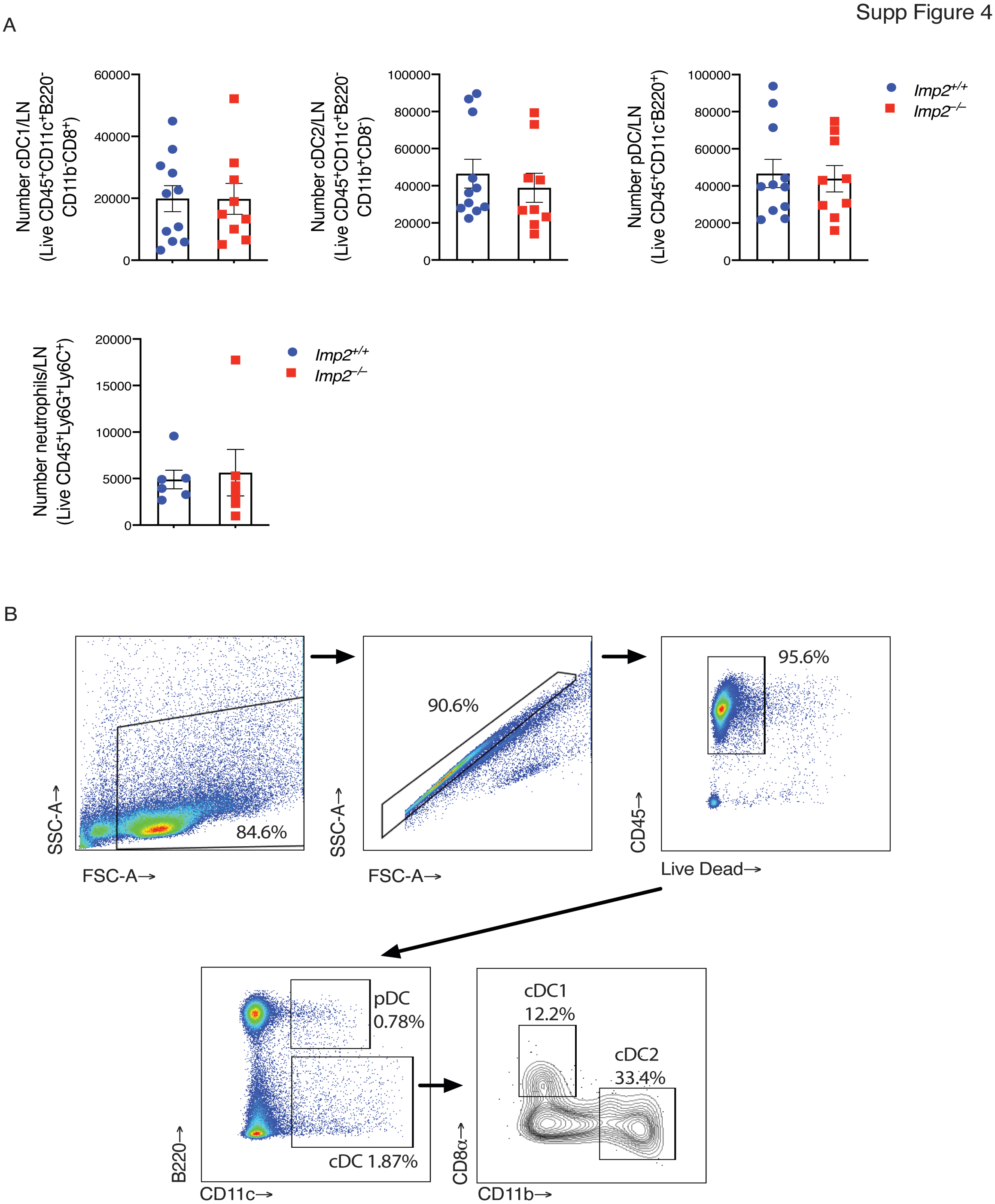

Supplementary Fig 4. Dendritic cells and neutrophils in Imp2$^{+/+}$ and Imp2$^{-/-}$ LN

Imp2$^{+/+}$ and *Imp2*$^{-/-}$ mice were subjected to EAE. Inguinal LN homogenates were prepared on day 7. (A) Live dendritic cells (DC) subsets were determined by staining for CD11c and B220, gated on the live CD45^{+} population. Live neutrophils were determined by staining for Ly6C and Ly6G, gated on the live CD45^{+} population. Graphs show numbers of cDC1, cDC2, pDC and neutrophils, pooled from 2-3 independent experiments. Each symbol represents one mouse. (B) Gating strategy used to characterize DC subsets.

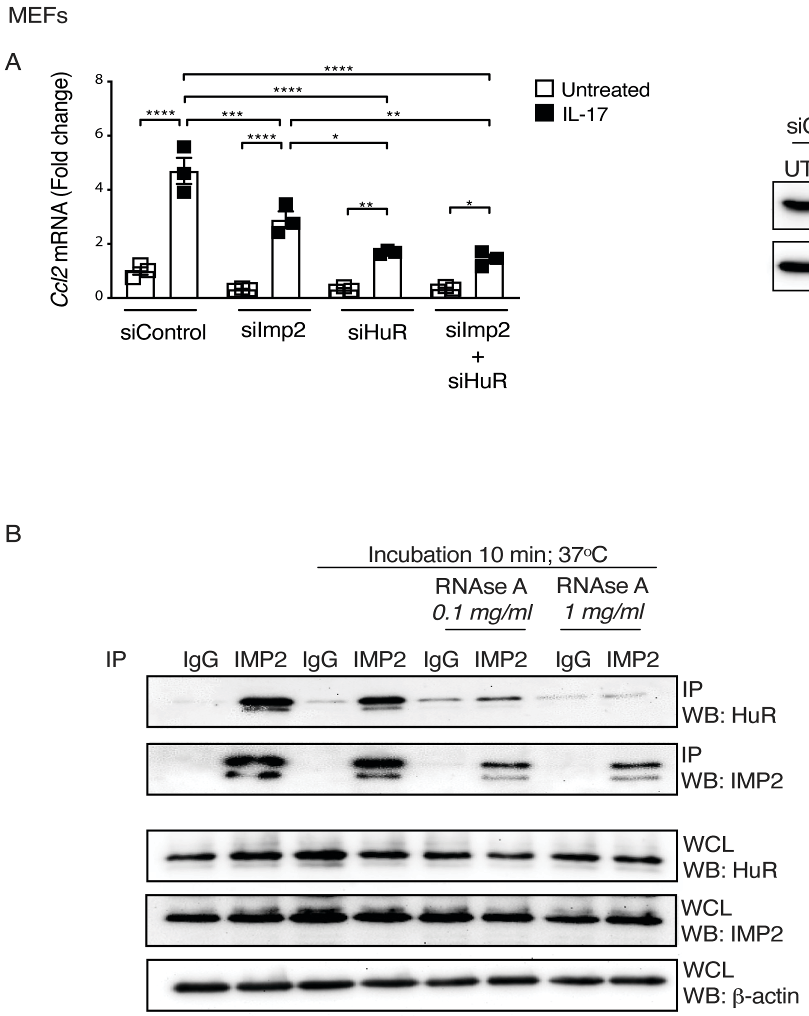

Supplementary Fig 5. HuR promotes IL-17-mediated Ccl2 expression

(A) MEFs were transfected with siRNAs targeting HuR or scrambled control and treated ± IL-17 for 8h. *Left*: *Ccl2* was assessed by qPCR normalized to *Gapdh*. Data are normalized to untreated samples with control siRNA and pooled from3 independent experiments ± SEM. *Right*: HuR protein expression in MEFs after knockdown was assessed by WB. (B) Lysates from MEFs either left untreated or treated with RNase A, were immunoprecipitated (IP) using anti-IMP2 Ab followed by Western blot analysis with the indicated antibodies. The data are representative of 2 independent experiments.
